# Gendered perceptions on infant feeding in Eastern Uganda: continued need for exclusive breastfeeding support

**DOI:** 10.1186/1746-4358-5-13

**Published:** 2010-10-26

**Authors:** Ingunn MS Engebretsen, Karen M Moland, Jolly Nankunda, Charles A Karamagi, Thorkild Tylleskär, James K Tumwine

**Affiliations:** 1 Centre for International Health, University of Bergen, Norway; 2 Bergen University College, Bergen, Norway; 3 Department of Paediatrics and Child Health, Makerere University, Kampala, Uganda; 4 Makerere University Clinical Epidemiology Unit, Kampala, Uganda

## Abstract

**Background:**

In resource-poor settings, HIV positive mothers are recommended to choose between 'Exclusive breastfeeding' (EBF) or 'Exclusive replacement feeding' (ERF). Acceptability, Feasibility, Affordability, Sustainability and Safety (AFASS) has been the World Health Organization (WHO)'s *a priori *criteria for ERF the last ten years. 'AFASS' has become a mere acronym among many workers in the field of prevention of mother-to-child transmission of HIV, PMTCT. Thereby, non-breastfeeding has been suggested irrespective of social norms. EBF for the first half of infancy is associated with huge health benefits for children in areas where infant mortality is high. But, even if EBF has been recommended for a decade, few mothers are practicing it. We set out to understand fathers' and mothers' infant feeding perceptions and the degree to which EBF and ERF were 'AFASS.'

**Methods:**

Eight focus groups with 81 informants provided information for inductive content analysis. Four groups were held by men among men and four groups by women among women in Mbale District, Eastern Uganda.

**Results:**

Two study questions emerged: How are the different feeding options understood and accepted? And, what are men's and women's responsibilities related to infant feeding? A mother's commitment to breastfeed and the husband's commitment to provide for the family came out strongly. Not breastfeeding a newborn was seen as dangerous and as unacceptable, except in cases of maternal illness. Men argued that not breastfeeding could entail sanctions by kin or in court. But, in general, both men and women regarded EBF as 'not enough' or even 'harmful.' Among men, not giving supplements to breast milk was associated with poverty and men's failure as providers. Women emphasised lack of time, exhaustion, poverty and hunger as factors for limited breast milk production. Although women had attended antenatal teaching they expressed a need to know more. Most men felt left out from health education.

**Conclusion:**

Breastfeeding was the expected way to feed the baby, but even with existing knowledge among mothers, EBF was generally perceived as impossible. ERF was overall negatively sanctioned. Greater culture-sensitivity in programs promoting safer infant feeding in general and in HIV-contexts in particular is urgently needed, and male involvement is imperative.

**Trial Registration:**

The study was part of formative studies for the ongoing study PROMISE EBF registered at http://clinicaltrials.gov (NCT00397150).

## Background

Recent studies suggest a growing awareness of the importance of partner HIV-testing and disclosure during pregnancies [1,2], and involvement of the father in prevention of mother-to-child transmission (PMTCT) programmes [3-6]. But to date, the area of maternal and child care in general and PMTCT in particular, has been characterised by limited inclusion of men in many African settings. HIV positive women are usually counselled on infant feeding options alone and have been left with the challenge of choosing the best or most appropriate infant feeding alternative. The choice the mother has had to make, often based on a newly conveyed HIV-positive message, is whether she should exclusively replacement feed (ERF) or exclusively breastfeed (EBF) her baby [7]. The problem is that even if she is perceived as the one to choose, she does not always control the conditions necessary for adherence to the option chosen. The discrepancies between how women are informed to feed their infants in the antenatal clinic and what is socially preferable in their communities represent major challenges [8].

Without any intervention, mother-to-child transmission risks were described as 30-45% in total: 5-10% during pregnancy, 10-20% during birth and 10-20% during breastfeeding [9]. Interventions became available reducing antenatal and intra-partum transmission by administering antiretroviral treatment during pregnancy and birth to the mother and postpartum to the infant [10,11]. Unfortunately, the benefits achieved were outweighed by the risk of transmission through breastfeeding. To eliminate the risk of HIV transmission postpartum the effect of free formula feeding from birth or after a period of breastfeeding were studied and non-beneficial effects were seen on survival [12-15]. Ten years ago South-African researchers described that EBF for six months carried just as low transmission risk as ERF [16]. The study was based on a smaller sample from a vitamin A trial. The results were later reconfirmed in other African settings [12,17]. Acknowledging the dilemma between the risk of HIV transmission through breastfeeding and the risk of communicable diseases through replacement feeding, a compromise was suggested by WHO: replacement feeding should just be practiced when 'AFASS.' The 'AFASS'-term reflected WHO's *a priori *criteria for replacement feeding: 'will replacement feeding be acceptable, feasible, affordable, sustainable and safe?' [18]. Among some workers in the PMTCT-field this term became a mere acronym. Different researchers have reflected upon the ethical dilemmas arising when programs present infant feeding-options as the mother's 'choice,' also in situations where neither of the five 'AFASS' criteria are fulfilled at the community level [19]. Before the 2006 guidelines were released, exclusive breastfeeding was recommended to be ended as soon as replacement feeding became 'AFASS' [18]. The 2006 guidelines changed focus and promoted exclusive breastfeeding for up to six months of life *unless *replacement feeding was acceptable, feasible, affordable, sustainable and safe for the mothers and infants before that time. At six months, if replacement feeding was still not acceptable, feasible, affordable, sustainable and safe, continuation of breastfeeding with additional complementary foods was recommended, while the mother and baby continue to be regularly assessed [20]. The 2010 guidelines repeat in content the 'AFASS' criteria, but has highlighted the need to 'use everyday language' to explain it [21]. Further, the content of AFASS is only applied to 'infant formula' and not other types of replacement foods such as diluted cow's milk taking into account the concerns with safety and nutritional value for long term use of it [22]. The focus now is how the HIV transmission risk can be reduced by either administering HAART (highly active antiretroviral treatment) to the mother from pregnancy throughout lactation or peri- and post exposure prophylaxis during breastfeeding to the infant [23]. Recent findings have made breastfeeding an even more attractive alternative for HIV positive mothers with transmission rates less than 1% observed in clinical trial settings in Africa [24-29]. These studies among others forced an update of the WHO guidelines towards increased use of antiretrovirals and combined breastfeeding in very low income settings. The newest guidelines also put a stronger burden of choice between EBF and ERF on the national and sub-national authorities which should guide the counselling of HIV-positive pregnant women. However, the women are still supposed to be informed about alternatives and make a choice. Even if the guidelines try to adapt contextual and ethical challenges the remaining problem exist with implementation and scaling up of programmes. In 2007, only 33% of the women living with HIV gained access to the PMTCT-services they should have had [30].

The feeding alternatives EBF and ERF apply in the context of mother-to-child HIV transmission, but promotion of EBF for the first six months of life *per se *is widely documented as an effective strategy for both short and long term health benefits for all children [31]. However, a recent pooled analysis from two African settings also suggests that there is no difference in HIV transmission risk between EBF and predominant breastfeeding where fruit juices and water based liquids are also allowed in addition to breast milk [32,33].

Both individual and group counselling have taken place to promote breastfeeding which mostly has included mothers [34]. Few studies exist on male involvement in the context of breastfeeding promotion, but a few studies exist from South-American contexts [35,36]. Recently, a Brazilian controlled clinical trial emphasized 'the need for increased cultural and behavioural understanding of the complexities determining infant feeding behaviour for the success of safer infant feeding promotion programs' [36]. To our knowledge there have been few studies in Africa addressing paternal involvement regarding infant feeding *per se*, and no studies in Eastern Uganda focusing on male involvement in infant feeding related matters by the time we conducted our study.

As part of the Integrated Management of Childhood Illness (IMCI) and Baby Friendly Hospital Initiatives (BFHI), EBF has been promoted for more than ten years [37]. These initiatives were based on comprehensive research documenting the benefits of EBF on infant mortality and morbidity [31,38]. Even with current knowledge about the benefits of exclusive breastfeeding, EBF-promotion has not been systematically and intensively implemented where infant mortality is high [39]. In addition, some of the programmatic investments made for promotion of EBF were confused by the HIV epidemic and some claimed a 'spill-over' effect from the PMTCT feeding guidelines were observed among the general population mothers [18]. Varying rates of EBF are therefore observed. Uganda Demographic and Health reports (UDHS) [40] found 50% of infants exclusively breastfed under six months based on 24-hour recall with a median duration of 3.1 months. EBF rates are higher for Uganda than in neighbouring East-African countries. In other studies from this area, peer support has been tried as a tool to increase the rates of EBF [41]. Still, a wider approach would be fundamental to avoid the mothers being solely responsible for her feeding choice and feeding capability, regardless of her HIV status. The question is whether promotion of 'exclusive breastfeeding' and 'exclusive replacement feeding' has been facing huge challenges because neither fulfils all the AFASS-criteria, not at individual or community level. This study explored how EBF and ERF were perceived among parents in communities in Eastern Uganda. It also explored the norms and social environment for infant feeding practices. Acknowledging the low adherence to the recommended feeding practices, both in the general population and in the HIV positive population in particular [42], we aimed at gaining deeper understanding of the complex patterns whereby decisions are made. Although both men and women were included in the study, our primary focus was on men as they are commonly left out in studies on infant feeding matters.

## Methods

The study was part of the formative research in Mbale, Eastern Uganda, that was conducted from 2003 to 2005 prior to a larger ongoing multi-centre trial PROMISE EBF: Safety and Efficacy of Exclusive Breastfeeding Promotion in the Era of HIV in Sub-Saharan Africa (http://clinicaltrials.gov/, Id no: NCT00397150). The results presented here are based on a total of eight focus groups discussions (FGD) among men and women in four sub-counties in Mbale District during October and November 2003.

### Study site

Mbale Municipality is situated approximately 200 km east of the capital Kampala at the foothills of Mt Elgon. The altitude is 1100 meters and the population about 76,500. Mbale Municipality is the main trading centre in Mbale district. The population is mainly Bagishu, but migration has produced large sub-urban areas with informal settlement and a multi-ethnic population. The so-called Industrial division in Mbale Municipality, was chosen as the urban setting for the study. Formerly this area had a lot of large factories, but it is now dominated by petty trading, carpentries, garages, bakeries and smaller industries. The rural setting of the study was Nakaloke, Namanyonyi and Bufumbo sub-counties in Bungokho county which surrounds Mbale Municipality. The rural population in the area depends on subsistence farming. All sub-counties except Namanyonyi had government health centres. Mbale Municipality had one district referral hospital. Smaller clinics were available in all sub-counties. The study participants had a maximum of one hour travelling by bus to reach the main hospital in Mbale. The site shared typical rural and urban characteristics for Uganda.

### Focus group discussions

Focus group discussions (FGD), which are described as appropriate arenas for discussing and airing multiple views on an issue, was chosen as the data collection method [43]. The respective views presented in FDGs do not necessarily reflect the participant's personal opinion, but rather his or her perception of what is the 'ruling attitude' [44]. Of the eight FGDs, one group of men and one group of women were recruited from each of the four sub-counties. Two men facilitated the discussions with the men, and two women facilitated the discussions with the women. The pair, one facilitator and one moderator, were Ugandan and fluent in the local language Lumasaaba. They had in-depth knowledge of infant feeding dilemmas in the context of HIV and university degrees in social sciences. They had former experience as FGD facilitators and were trained additionally for this particular study. Throughout the data collection period, they were closely followed-up by the study team (CAK and IMSE). The recorder took notes and observed body language and the general atmosphere in the groups. All interviews were tape recorded after consent was given. Paper transcripts were used as back-ups. The participants were recruited by the local vaccinator or local chairman (LC) in their respective villages. The inclusion criteria were 'having children younger than 5 years' and 'give informed consent for participation'. Among altogether 81 informants, 38 were men and 43 were women. Of these, 10 men and 11 women were urban dwellers. The participants were mainly subsistence farmers and petty traders and their reported age ranged from 16 to 40 years. The men and the women participating were not related as couples. Their HIV-status was not known to the study team. The venues chosen for the discussions did not have any link to a health institution. Confidentiality within each group discussion was agreed among the participants prior to the start. Ethical clearance was obtained from Makerere University Faculty of Medicine Ethics and Research Committee.

### Analysis

The following topics were investigated in all eight groups using a focus group discussion guide with open ended questions and numerous probing alternatives: (1) knowledge of breastfeeding, cultural norms and early breastfeeding practices; (2) reactions and feelings about non-breastfeeding; (3) support and responsibility for child rearing and feeding priorities within the family; (4) child nutrition and nutrition related morbidity; and among men only: (5) satisfaction with the communication about child nutrition targeting men at the local health units. Each discussion lasted approximately 2.5 hours.

Data were transcribed before translation into English by those who conducted the discussions. The findings were discussed with the principal investigator (IMSE) after each FGD. The principal investigator (IMSE) also explored areas for further probing and decided on the time of saturation. Inductive content analysis was performed [44]. Reading was done in consecutive sessions. The coding was done phrase by phrase and manually cut and pasted in categories before broader themes emerged. The end result was compared to previous pre-analysis. The analysis was done by the first author in collaboration with the second author (IMSE and KMM). The two major themes identified were: 1) feasibility of existing infant feeding recommendations; and 2) gendered differences regarding infant feeding. A potential strength of the study was that men and women were kept in separate groups during the discussions. This might have increased openness and sharing amongst both men and women. A potential weakness was the single method strategy adopted utilising only focus group discussions and the language barriers existing between the analysis team and the study participants.

## Results

Based on the identified themes two questions emerged to guide the presentation of results. First, how are the different feeding methods, EBF and ERF understood and accepted? Second, what are men's and women's responsibilities related to infant feeding?

### How is breastfeeding understood?

#### Initiation of breastfeeding

The attitudes towards initiation of breastfeeding in general, and colostrum in particular, varied. Some of the men were aware of the value of colostrum and recognised it was good and nutritious for the newborns. Others held the view that it was necessary to discard the first milk. One reason mentioned for squeezing it out, was to avoid 'blocking' of the breasts. More commonly reported was the belief that colostrum could *'cause diarrhoea,' 'contained dirt' *and was *'bad*' for the baby.

But, even if the intention to start breastfeeding immediately after delivery was there, *'delayed milk*' or *'lack of milk*' and other difficulties were reported by men and women as main reasons for delayed initiation. The women had experienced stomach pain, loss of blood, delivery of placenta, exhaustion, stitching and even collapse and unconsciousness as factors delaying initiation of breastfeeding. Furthermore, a sleeping or sick newborn baby was reported as obvious reasons to delay breastfeeding, the baby not being capable of suckling.

Ideas about the importance of a clean body in general and clean breasts in particular could contribute to delays. Bathing mother and baby and washing of breasts were mentioned in all female groups as an important task to do before breastfeeding could start. This practice was also observed by men.

'I first have to bathe the baby then myself before I begin breastfeeding.' (Urban FGD Namatala, woman)

Skilled attendants during delivery also involved washing and wrapping prior to breastfeeding.

'The midwife first stitched me; I bathed and then put the baby on the breast.

'The midwife had to weigh and wrap the baby, and I also had to recover since I bled a lot.' (Urban FGD Namatala, women)

In exceptional situations where it was hard to explain why breastfeeding was not established as expected, some of the women mentioned supernatural forces like spirits, curses and bewitching.

'I heard that there are some spirits that stop the baby from breastfeeding, so they must please them first by offering sacrifices and give a name before the breastfeeding starts.' (Rural FGD Nakaloke, woman)

Name giving as a ritual prior to breastfeeding could involve drops of pre-lacteals like water or local brew. Both rural and urban female groups referred to this tradition.

'Some people believe that an elder has to give a name to the child before the mother breastfeeds. He puts his fingers in water, and then puts it in the baby's mouth before the mother breastfeeds.' (Rural FGD Namanyonyi, woman)

One female group also raised the issue of sexual taboos. The breasts of a nursing mother should not be sucked as part of a sexual act, lest it may harm the infant:

'If a husband suckled the breasts of the mother, the baby she gives birth to is not supposed to breastfeed or else the baby dies.' (Rural FGD Bufumbo, women)

Pre-lacteal feeding was perceived as common and closely linked to perceived lack of milk and delayed initiation of breastfeeding. Men mentioned *'warm water' *or *'diluted fresh cow's milk*' as pre-lacteals. Warm water should help with *'unblocking' *the baby's throat before other feeds could be given. The women mentioned *'plain water,' 'glucose water,' 'sugar and/or salt and water' *and *'Gripe water' *as pre-lacteals. A few women specified that they boiled the water.

One reported influence from her own relative:

'My mother stopped me from giving breast milk unless I first gave sugar water.' (Rural FGD Nakaloke, woman)

The mothers also gave pre-lacteals because the *'baby was crying,' 'born hungry,' 'needed energy quickly,' 'that the breast milk was too light' *and they had *'pain' *and were *'exhausted.' *Breast milk given first was also believed to be bad among some women.

'Milk brings worms in the stomach therefore I first give glucose water.' (Rural FGD Nakaloke, woman)

#### The importance of breastfeeding: the only way to feed an infant

Breastfeeding had a central position in the local infant feeding culture. Men described breastfeeding in general as '*the only way to feed*' an infant and regarded breastfeeding entirely as a mother's duty. This view about breastfeeding was rooted in traditions.

'It is their tradition that their grand parents took on, so they too have taken it up, the mothers have to breastfeed as well as feed just like the grand parents did.' (Rural FGD Bufumbo, man)

But even if the men took breastfeeding for granted, they also argued for the developmental assets of breastfeeding: *'physical growth,' 'intellectual' *and *'psychological development' *were mentioned in the male groups as important reasons for the child to receive breast milk. In addition, a few mentioned it contained the *'necessary nutrients' *for the baby and was a *'source for energy' *for the babies who *'have no teeth for chewing' *and stomachs which *'are not ready.' *The preferred time to stop breastfeeding was between one and two years according to the men, or later if the child wanted, but depended most on when the mother conceived again.

'It depends on the time the mother takes to conceive, this is the time the children are weaned off to give way for another one.' (Rural FGD Bufumbo, man)

#### Ideas about exclusive breastfeeding

Although the men strongly argued that breastfeeding was a condition for child survival, they were generally unfamiliar with the idea that an infant should be breastfed exclusively for the first six months. They complained that they did not have access to infant feeding teachings and hence, *'had learnt nothing' *about it. Even if a few men did describe EBF as *'the only way' *and *'the easiest way' *in the first couple of weeks after birth, there was a general agreement among the men that breast milk was insufficient food for the baby.

*'Breast milk alone cannot give the baby sufficient energy *- *that's why other foods are introduced as soon as possible.' (Urban FGD Namatala, man)*

The women by contrast had been taught about EBF in antenatal clinics. They were familiar with the recommendation to breastfeed exclusively for six months, and they were aware of the immunological and nutritional values of breast milk in general and EBF in particular. Nevertheless, the women also expressed a general scepticism to the concept of exclusive breastfeeding and were worried that they did '*not have enough' *or had '*no milk*.'

They were confused and uncertain about the recommendation to give '*nothing, but breast milk*' for six months and did not understand the rationality behind it. The following quote grasps some of the frustration related to it:

'I want to know why they refuse us to give other feeds during the first six months'. (Rural FGD Nakaloke, woman)

The barriers to EBF were multi-faceted. In addition to the concern about insufficiency, some women expressed that EBF could be harmful and could cause sickness of various kinds in the baby like *'wide stomachs' *and *'worms'. *In this context breast milk supplements were described as beneficial and would make the baby *'more healthy,' 'immune,' *and *'fatter' *and would help *'to avoid worms*.'

More important was the experience that EBF was very time-consuming and difficult to practice for a mother who also had other commitments in the household or outside. A woman's chores in and around the house including cooking, cleaning, collecting water and firewood as well as taking care of the children and elders were described as so demanding that it made EBF difficult:

'I have a lot of work at home so I have no time to breastfeed exclusively.'(Urban FGD Namatala, woman)

Women described working outside the home and being employed as virtually impossible to combine with exclusive breastfeeding:

'Some working mothers have no option but to introduce other feeds.' (Rural FGD Namanyonyi, woman)

Quite a few of the participants were still in school and said they had no time to breastfeed or at least could not breastfeed exclusively. The mother also needed to be relieved from the stress of a crying baby.

'The baby has to get satisfied so that he does not cry' (Urban FGD Namatala, woman)

The vulnerability involved in depending on breast milk only was expressed through the concern that' *the baby should get used to other feeds*'. In addition, giving supplements to breast milk was seen as a way to solve practical challenges so the women could earn money. Some of the urban men were bottle-feeding their infants during the discussion because their wives were studying or working. The rural men assigned too early introduction of supplementary foods to the *'working class women' *contrasting with the *'rural women' *who did it later. On the other hand, in the rural context, availability of cow's milk could in itself be a reason to introduce supplements. *'I have a cow' *was mentioned by a few men and women as a reason to give cow's milk.

Most importantly, men and women were concerned about breast milk not being sufficient and EBF as not feasible in their particular poverty-ridden environment. Sickness and hunger were held as major reasons for poor milk production and hence for giving other foods:

'Sickness like malaria and breast problems like breast engorgement which are very common here do not allow the mothers to breastfeed exclusively.' (Urban FGD Namatala, man)

The exhaustion following HIV/AIDS was mentioned as a reason to introduce other foods early and thus practicing mixed feeding:

'Sickness like HIV/AIDS stops some mothers from breastfeeding exclusively.' (Urban FGD Namatala, woman)

The women explicitly linked their perceived low breast milk production to poor food intake saying that *'because mothers feed poorly they don't get enough milk' (Rural FGD Nakaloke, woman). *Poverty and hunger were major concerns:

'Poverty makes me fail to buy food and so I don't eat a balanced diet which limits the milk for the baby' (Rural FGD Nakaloke, woman)

In a context where the men were seen as the main providers, women tended to hold men responsible for the scarcity of food and hence for their poor milk production:

'Husbands do not provide enough; most times they only provide millet bread, so there is no milk in the breast.' (Rural FGD Namanyonyi, woman)

These views were to some extent supported by the men who saw it as their obligation to contribute with extra food for the postnatal breastfeeding woman. Both men and women stressed that neglect; lack of food and care during the important period of breastfeeding, could cause emotional problems that again could affect breastfeeding negatively.

'Emotions cause less milk, especially if the husband is not looking after the mother well.' (Rural FGD Bufumbo, woman)

The link between poverty and poor ability to practice EBF was not unanimously held. While women said they were too poor to practice EBF because it demanded good food, men tended to associate EBF with poverty and lack of ability to buy complementary food.

*'Exclusive breastfeeding exists because of financial constraints *- *one's income may be too low to attain other foods. (Rural FGD Nakaloke, man)*

#### How is non-breastfeeding understood and accepted?

The possibility of a mother not breastfeeding her newborn was received with surprise and disdain. Some women mentioned the possibility that the milk could turn *'bad' *with sickness like swollen breasts, cancer, HIV/AIDS and malaria, but in general avoidance of breastfeeding was an unexpected behaviour. A non-breastfeeding mother was looked upon with suspicion. Her reasons would commonly not be taken seriously:

'Some just pretend to be sick and they refuse to breastfeed.'(Rural FGD Nakaloke, woman)

Women in all groups were concerned that some women would prioritize their own appearance over the health of the child:

'Others want to remain young and want breasts to remain firm.'(Rural FGD Namanyonyi, woman)

Men and women shared the understanding that avoidance of breastfeeding would severely harm the baby. As one man put it:

'Such a mother will be taken as one who does not understand because she will be denying the baby a chance to grow well.' (Rural FGD Namanyonyi, man)

Denying an infant breast milk was associated with the death of the child and the mother would be held responsible. A mixture of feelings including moral judgement and disgust were seen among men and women alike. One of the men said that such a woman '*would be taken as a murderer' (Rural FGD Namanyonyi, man). *The women used words like: *'difficult,' 'not serious,' 'irresponsible,' 'not ready for her baby,' 'abnormal,' 'mentally disturbed,' *'*bad hearted' *and *'wants her baby to die' *about non-breastfeeding mothers. A strong social judgment was observed. A mother who did not breastfeed did not deserve the status of a mother. As one woman expressed it: *'She is not supposed to be among mothers' (Urban FGD Namatala, woman)*

Apart from the acute survival issue involved in not breastfeeding an infant, the women were concerned about the emotional aspects of breastfeeding. Some felt sorry for mothers who did not breastfeed.

'I feel sorry for her because she does not know the importance of breastfeeding.' (Rural FGD Nakaloke, woman)

Even more, they were worried about the bonding between mother and infant.

'It's a sad moment because there is loss of love and intimacy between the mother and the baby.' (Urban FGD Namatala, woman)

A decision not to breastfeed on the part of the mother was perceived as an action that would require intervention on the part of the father. Unless a mother had a good reason to abstain from breastfeeding, like psychological or psychiatric conditions or lack of breast milk, the sanctions, according to the men could be rather tough.

'I would force her to breastfeed unless she has a genuine reason like serious mental illnesses.' (Urban FGD Namatala, man)

Some men said they would first just ask why. Others would *'plead her because it looks so bad.' *If they were the father of the child some would try and find immediate solutions like feeding the baby with an alternative feed. Porridge with margarine, cow's milk, warm water and wet nursing were suggested. None of the men imagined they would sustain this for a long period of time, neither economically nor practically. Others would seek assistance from relatives or health workers. Physical force was mentioned by a few. One man said: he *'can even beat her.' (Rural FGD Bufumbo, man)*

In all groups men said they would rely on the elected administrative person in the village (local chairman, LC) or some other person with administrative or judicial responsibilities.

'Such a mother will be taken either to probation offices, LCs [local chairman], or police immediately.' (Rural FGD Bufumbo, man)

Some expressed that a non-breastfeeding decision on the part of the mother would result in divorce.

'I would report her to the LCs [local chairman] and she will cease being my wife.' (Urban FGD Namatala, man)

In general, not breastfeeding an infant was seen as a serious neglect of maternal responsibility and was sanctioned accordingly.

## Discussion

This study has documented the important position of breastfeeding in the infant feeding culture of Eastern Uganda and has thrown light on breastfeeding as the only accepted way to feed an infant. Furthermore, it has illuminated the numerous problems associated with the concept of 'exclusive breastfeeding' which was generally rejected as a feasible and sufficient infant feeding method for the first six months of life among the study participants. The study revealed that not breastfeeding was highly unacceptable and would attract severe social sanctions except in cases of serious mental or somatic maternal illness. Based on the perceived threat for the baby, men commonly saw it as their duty to implement sanctions in case the mother decided not to breastfeed.

In the WHO HIV and infant feeding guidelines the AFASS-term has been linked with ERF. Based on scientific evidence of its benefits, the latest guidelines promote EBF much stronger than earlier. Benefits of EBF have been seen both on HIV-free survival and on mortality reduction as a whole [12]. EBF can therefore be recommended irrespective of HIV status, but EBF remains uncustomary and low rates of EBF have been reported worldwide. This study has addressed not only the AFASS of ERF, but also to what extent EBF is acceptable, feasible, affordable, sustainable and safe. This study revealed that: EBF was only partially *acceptable *and often perceived as associated with reduced growth and increased sickness. Among women, EBF was regarded as extremely difficult to combine with domestic chores and it was seen as an obstacle to pursuing income-generating activities. Most women went back to work after one or two months. Hence, very few mothers *sustained *EBF for a long period. Only women well provided for could afford to practice EBF for six months. Furthermore, women felt that poverty and hunger reduced their breast milk production. These views were supported by men, but additionally men considered EBF as a sign of poverty and as a sign of them failing to provide food for the infant. Many women had learnt that EBF for six months was *safe *and nutritionally adequate, but others doubted these messages. In order to improve the overall adherence to EBF among mothers in the general population these issues need to be addressed.

Previous studies have described 'stigma' associated with replacement feeding and have argued that ERF is not 'AFASS' in communities where breastfeeding has been normative [7]. The present study has added knowledge about the link between breastfeeding and motherhood and argues that in the study area, breastfeeding is inseparable from, and cannot be understood without, an appreciation of what constitutes motherhood. The description of non-breastfeeding mothers as '*murderers' *and '*she is not supposed to be among mothers' *must be understood in this context. Hence ERF was not acceptable, feasible, affordable, sustainable or safe.

The study revealed that recommendation about 'exclusive breastfeeding' for the first six months was known to women, but also that it was poorly internalised. The quote: '*I want to know why they refuse us to give other feeds during the fist six months' *illustrates how women had learnt the public health message, but had not fully understood it. This view was present among most women in all the female groups. Different explanations are possible: (1) The women's need for internalised knowledge could reflect difficulties and weaknesses on the system level. A few studies within the PMTCT-context suggest that misinterpretation and lack of knowledge among health workers is prevalent [45]. Another recent study suggests that challenges exist for the health workers with 'clients, staff, infrastructure and existing healthcare' [46]. (2) The need for internalised knowledge could also reflect reduced communication on the individual level. Teaching from government staff has been shown to be less efficient than peer-counselling [31]. A qualitative study from Uganda suggests promising conditions for peer counselling [41]. (3) The women expressed that they did not have enough to eat because they were poor so they could not produce enough milk. Women felt they were constrained in their daily life: obligations, exhaustion, studies, domestic work, employment and family pressure were factors which made EBF difficult. It is unlikely that any programs will be successful in promoting EBF unless women feel that these factors are taken into consideration.

This study raises the question of whether understanding the complexities of achieving EBF has been missing or not fully addressed on program level. Mixed feeding patterns as the ruling infant feeding pattern has been described for a long time in many different sub-Saharan African countries including Uganda [47-49]. In 1991 UNICEF launched the Baby-Friendly-Hospital Initiative, including 'Ten steps to successful breastfeeding'[50]. According to the UNICEF evaluation from 2002, 11 hospitals in Uganda, including Mbale referral hospital were considered so-called 'baby-friendly,' but limited resources compromised the full services required for 'baby-friendly' status [51]. Further, in 1995 the WHO initiative 'Integrated management of childhood Illness (IMCI)' was launched as a comprehensive package to address the most common diseases being responsible for child mortality [52]. Uganda was one of the first countries to adapt this package with three test-districts in 1995, and 55 out of 56 districts had implemented IMCI by 2000 [53]. Mbale district hospital also took up the PMTCT-programme to one of its tasks in 2000. These large programs have been important tools to streamline comprehensive packages where nutrition and infection aspects are taken into account. Recently, an evaluation has been ongoing (Multi-Country Evaluation) where Uganda was one of the test-countries [53]. Even if there are obvious benefits to the programmes, some researchers have been commenting on the limited effect we have seen from the programmes on reduction of the prevalence of mixed feeding during the first six months so far [54]. Many qualitative studies have explored mothers' reasons for mixed feeding, and as this one, found a myriad of cultural, practical and personal explanations for women to practice mixed feeding [55-58]. Even if larger top-down programmes like BFHI, IMCI and PMTCT - strategies may set the standard for teaching, follow up and recommendations, the question is whether further supportive strategies are needed for better infant feeding practices to be adopted at community level. Peer support could be one such strategy [39] or expansion of community health worker services could also be further explored. Obstacles to improved EBF trends might also involve societal factors such as regulations regarding maternal leaves, media and the implementation of 'the code of marketing'[59] and women's and children's status affecting public breastfeeding. These types of barriers must be addressed in parallel with implementation of programmes.

Comprehensive postnatal research has been conducted in Ghana describing improved early infant feeding practices as key to reduce neonatal mortality [60]. A recent qualitative assessment from Ghana found 'perception of a lack of breast milk', 'performing post birth activities such as bathing,' 'perception that the mother and the baby need rest after birth' and 'the baby not crying for milk' as reasons to delay initiation of breastfeeding [58]. These findings are consistent with the findings in this study about initiation of breastfeeding. Elimination of these barriers could potentially reduce the proportion of women initiating lactation later than recommended in settings like that of Uganda and Ghana, and enhance early and exclusive breastfeeding.

This study found that male participants were dissatisfied with the health system's neglect of men. Most men had strong feelings about the importance of breastfeeding for the survival of their children. A non-breastfeeding decision was therefore a potential threat to him as a parent. Anger leading to sanctions must be understood in this context. The male participants had limited knowledge about EBF and its potential benefits. Inclusion of fathers during pregnancy and postpartum health care could therefore improve the acceptance of current infant feeding guidelines including EBF. Strategies used by health workers in this context was not sufficient, they invited the men by giving messages to the women, but the men did not come.

Quantitative studies in this region, both in the communities and among HIV-positive mothers, were done prior to this qualitative analysis and they confirm parts of the findings of this study with high proportion of mothers giving pre-lacteals, low rates of EBF and insufficient supplementary feeds with effect on growth outcomes. In addition, huge challenges were seen regarding infant feeding in the field of PMTCT [47,61-63]. This study elaborated further and tried to understand the rationale behind the observed practices and reactions to the different feeding options. The results from this study are also supported in the literature from other sites [54,58]. But, as this study documents, there are also other obstacles experienced by parents that need to be further addressed in order to succeed in the promotion of EBF. The immediate implication from this study is that there is a knowledge gap between what the mothers have perceived from their maternity education and their actual knowledge which should be bridged. One major message which comes out of this study is that we cannot succeed in the promotion of EBF unless men are targeted in health education and made co-responsible for infant feeding. Much could be gained for their partners if the men were brought on board in child rearing. New strategies need to be developed and tested to accomplish that. Lastly, a wider community involvement and political will is needed to strengthen the rights for a woman to practice her chosen infant feeding option. Today both EBF and ERF are experienced as practically unfeasible and culturally unacceptable to parents. Further political commitment including job and study security during maternity leave is an urgent issue to address.

## Conclusion

It is a concern that underlying socio-cultural mechanisms to recommended infant feeding practices in the communities are not addressed to a larger extent among program implementers. In this study area, breastfeeding was seen as the only way to feed the baby, but even with existing knowledge among mothers about recommended feeding practices, EBF was generally perceived as not feasible or even harmful. ERF was socially unacceptable and perceived as a threat to infant survival, especially among men, and could cause severe sanctions. Involvement of fathers in infant feeding training is imperative for increased acceptance of recommended infant feeding practices, EBF in particular, in the Eastern Ugandan communities studied.

## Competing interests

The authors declare that they have no competing interests.

## Authors' contributions

IMSE was active during the design, implementation, analysis and writing. KMM and JN contributed to the analysis and writing. CK contributed to the design and implementation and JKT and TT contributed to the design and intellectual content. All authors read and approved the final manuscript.
